# Failure Analysis of a Novel Ceramic-Coated Floating Oil Seal Considering O-Ring Initial Assembly Deformation

**DOI:** 10.3390/ma18194592

**Published:** 2025-10-03

**Authors:** Yuehao Zhang, Fengsen Wang, Zhumin Li, Bozhao Sun, Tianci Chen, Jiao Wang

**Affiliations:** 1 Engineering Training Center, Yantai University, Yantai 264005, China; yuehao@ytu.edu.cn; 2 School of Electromechanical and Automotive Engineering, Yantai University, Yantai 264005, China; 13361248974@163.com (F.W.); 19861106553@163.com (Z.L.); 18333257521@163.com (B.S.); 15650443171@163.com (T.C.)

**Keywords:** ceramic-coated floating oil seal, O-ring, consideration of assembly process, failure mechanism analysis

## Abstract

The floating oil seal (FOS) is a critical component in coal mining machinery, where frictional wear and high stress on the O-ring can lead to oil leakage and eventual FOS failure, significantly impairing equipment performance. To address this issue, this study proposes a novel ceramic-coated floating oil seal (NCCFOS) composite structure that enhances wear resistance without modifying the existing sealing cavity configuration. A two-dimensional axisymmetric finite element model of the NCCFOS was developed based on the Mooney–Rivlin constitutive model, considering the O-ring assembly process for improved accuracy. The model was analyzed under oil pressure loading, with parametric studies examining the influence of oil pressure, assembly clearance, and material hardness on O-ring stress, contact pressure, and frictional stress distribution in the floating seal ring. The results demonstrate that accounting for the assembly process yielded more realistic stress predictions compared to conventional modeling approaches. The NCCFOS design effectively mitigated stress concentrations, reduced O-ring wear, and extended fatigue life, offering a practical solution for enhancing the reliability of coal mining machinery seals.

## 1. Background & Summary

The floating oil seal (FOS) is a widely adopted and extensively studied sealing solution for shearer output shafts, renowned for its robust performance and long-term durability, which critically influence shearer operational efficiency [1,2]. However, the hyperelastic O-ring, which serves as a key yet vulnerable component of the FOS, significantly compromises sealing performance under dynamic loading conditions despite the system’s overall reliability [3]. Recent investigations have increasingly identified surface friction and wear of the O-ring as a predominant failure mechanism in floating oil seals, leading to premature leakage and functional degradation [4].

Current research on O-rings is mainly focused on finite element simulation techniques [5,6,7,8]. Green et al. [9] systematically investigated the axial and radial compression characteristics of O-rings and analyzed the effect of groove geometry and friction on sealing performance. Zhang et al. [10] considered the fluid pressure factor and used ANSYS Workbench 2019 software to analyze the dynamic equivalent stress and contact stress of O-ring components under fluid pressure load and piston liner oscillation load, thereby determining the specific location and cause of failure. Zhang et al. [11] focused on quantifying the effects of precompression magnitude, fluid pressure, and friction coefficient on static and dynamic sealing performance. Zhou et al. [12] evaluated the sealing integrity of rubber O-ring/wedge-ring composite structures in ultra-high-pressure gaseous hydrogen environments. Hu et al. [13] analyzed the influence of typical parameters such as the gap between the upper and lower flange openings, initial compression, sealing groove and groove bottom chamfer radius, sealing groove width, O-ring material, O-ring cross-sectional size, and working temperature on sealing performance. Chen et al. [14] used numerical simulation to analyze the influence of O-rings on the end face deformation of mechanical seal flexible rings. Wu et al. [15] established a nonlinear FEM revealing that extreme environmental pressures trigger volumetric shrinkage and compressional deformation in O-rings, leading to significant sealing degradation. El Bahloul et al. [16] identified three material-dependent reliability determinants: seal compression ratio, hardness, and friction coefficient under varying pressures. Shi et al. [17] demonstrated that side pressure variations exerted greater influence on equivalent stress than contact pressure or friction coefficient changes in FOS structures, highlighting critical design considerations. Huang et al. [18] combined experimental and simulation approaches to analyze seal failure mechanisms. Zhang et al. [19] used the Contour GT-K to analyze the wear morphology of the sealing surface, determined the contact pressure distribution, and evaluated the effects of drilling fluid pressure and high temperature using the finite element method. Zhao et al. [20] used finite element simulations to compare textured and non-textured surfaces, revealing the influence mechanism of surface textures on friction behavior. He et al. [21] systematically compared geometric textures (square, 2:1 rectangular, triangular, hexagonal, circular), demonstrating that controlled roughness parameters (depth, angle, area ratio) significantly improved sealing performance. Merkle et al. [22] developed an adjustable heating and cooling test platform to evaluate radial seals. Their study demonstrated that variations in temperature significantly influenced wear behavior.

However, current finite element analysis methodologies for FOS exhibit significant limitations, particularly in their failure to account for the structural deformation of floating seal seats following O-ring installation. This oversight leads to substantial inaccuracies in simulating the O-ring’s true positional relationship within the seal assembly during actual operating conditions. While recent advancements have enhanced the sealing performance of FOS systems, wear-induced failure continues to pose a major reliability challenge. Therefore, reducing the maximum VMS, contact friction, and contact pressure on the O-ring, and enhancing its wear resistance are crucial. In this paper, through experimental research and simulation analysis, an in-depth study was conducted on both FOS and NCCFOS. The study explored the effects of oil pressure, assembly clearance, and hardness on O-ring stress, contact pressure, and contact friction stress of the O-ring, providing theoretical and experimental support for developing more reliable FOS.

The framework of the paper is as follows. In Section 2, the constitutive equation of the rubber material is established, and the corresponding parameters for different hardness levels are calculated. FEM of the NCCFOS is established, and the relevant parameters for nonlinear contact analysis are defined. Section 3 details the parametric studies performed to evaluate the effects of oil pressure, assembly clearance, and material hardness on performance metrics of the floating seal’s O-ring, specifically its stress, contact pressure, and frictional stress distribution. In Section 4, the findings have been summarized in the conclusion.

## 2. Methods

### 2.1. Constitutive Equation of Rubber Material Constitutive Model

**Figure 1:**
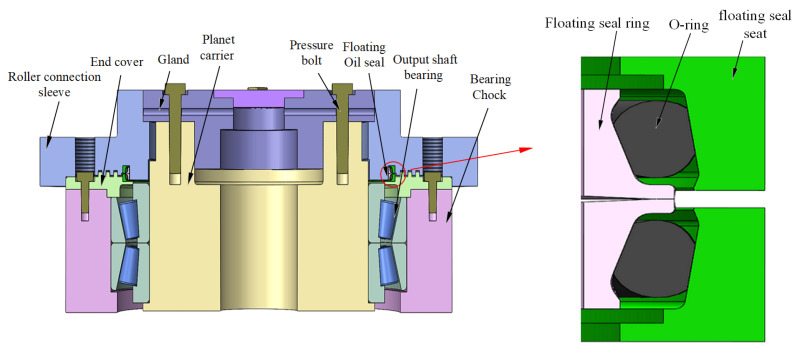
Schematic of the floating oil seal on the coal mining machine rocker arm.

The FOS comprised a floating seal ring, O-ring, and floating seal seat. Its structural diagram was developed in SolidWorks 2022 and is shown in Figure 1.

The O-ring is primarily composed of rubber, a super elastic material exhibiting both complex material nonlinearity and geometric nonlinearity. This study employed nitrile rubber, whose constitutive relationship is commonly described by the Mooney–Rivlin model. The two-parameter form of the Mooney–Rivlin constitutive equation is expressed as follows [23,24,25,26].

(1) $$W=C_{10}(I_{1}-3)+C_{01}(I_{2}-3)$$

where W denotes the strain energy density; $I_{1}$ and $I_{2}$ represent the first and second deviatoric strain invariants; and $C_{10}$ and $C_{01}$ are the Mooney–Rivlin material constants.

The relationship between modulus and hardness of rubber is [27]:

(2) $$E=\frac{15.75+2.15\mathrm{HSA}}{100-\mathrm{HSA}}$$

where HSA is shore hardness, and *E* is the modulus.

(3) $$E=6(C_{10}+C_{01})$$

$C_{10}$ and $C_{01}$ satisfy the following relationship according to the empirical formula:

(4) $$C_{01}=0.25C_{10}.$$

The uncompressible constant *D*_1_ is:

(5) $$D_{1}=\frac{1-2\mu }{C_{10}+C_{01}}.$$

where μ is the Poisson ratio whose value is 0.499.

Based on Equations (2)–(5), the material parameters for rubber at different hardness levels are presented in Table 1.

### 2.2. Finite Element Modeling of the Ceramic-Coated Floating Oil Seal

**Figure 2:**
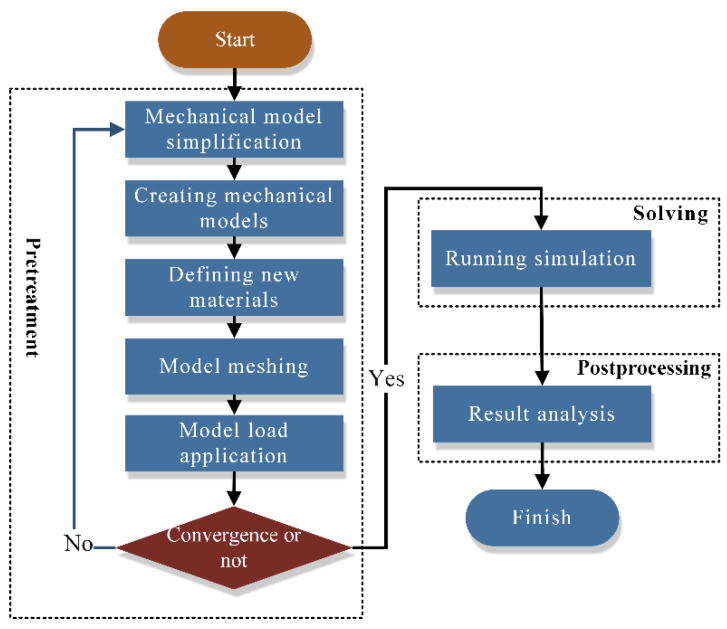
Floating oil seal finite element analysis process.

Figure 2 illustrates the finite element analysis (FEA) process for the FOS. The finite element method (FEM) is employed to simulate FOS performance under various operating conditions. The FOS geometry is first converted to a FEM with applied boundary conditions and loads. Numerical methods are then used to determine the stress distribution, deformation, and O-ring sealing performance, with the goal of optimizing design and material selection.

**Figure 3:**
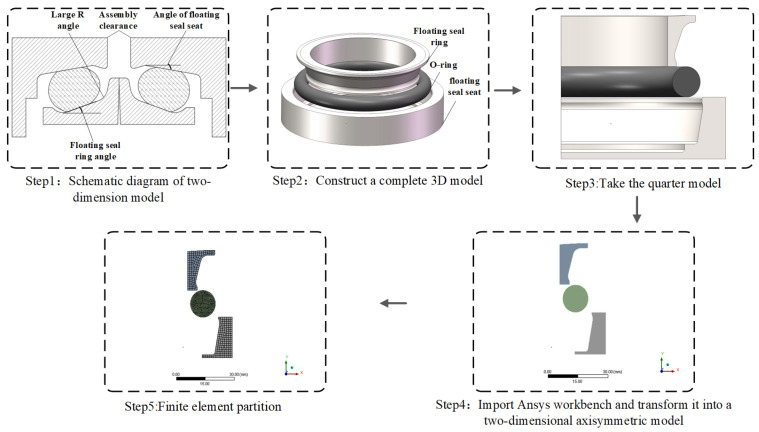
FOS modeling process.

**Figure 4:**
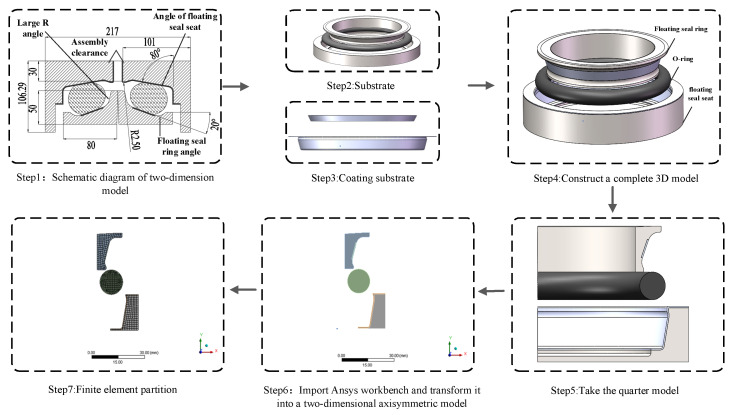
NCCFOS modeling process.

The floating seal seat and ring were fabricated from high-carbon chromium bearing steel (GCr15), exhibiting an elastic modulus of 210 GPa, density of 7850 kg/m^3^, and Poisson’s ratio of 0.3. Figure 3 illustrates the FOS modeling process. During operation, external mud ingress reduced surface smoothness of the seal ring and seat, impairing conical surface coaxiality relative to the reference shaft and accelerating O-ring aging. To enhance wear resistance and mitigate O-ring stress concentration, a novel FOS design was developed (Figure 4).

Addressing potential O-ring extrusion, alumina ceramic plates were embedded into the inner walls of both seal components at O-ring interfaces. These plates possessed an elastic modulus of 376 GPa, density of 3700 kg/m^3^, and Poisson’s ratio of 0.3. Contact pairs were defined between: (1) the floating seal seat and O-ring, and (2) the floating seal ring and O-ring. In these pairs, the O-ring surface served as the contact surface, while the seal components acted as target surfaces. For the NCCFOS configuration, the target surfaces additionally included the ceramic layer. Both frictional (coefficient μ = 0.2 for metal O-ring interfaces; μ = 0.06 for ceramic O-ring interface) and bonded contacts (between ceramic layer and seal components) were implemented. Detailed FEM settings are provided in Table 2.

When analyzing and calculating, fixed constraints were applied to the bottom end and right side of the floating seal seat to prevent displacement in the X and Y directions (refer to Step 5 in Figure 3). The left side of the floating seal ring was constrained by 0 displacement in the X direction to ensure the stability of the X direction. The displacement constraint in Y direction was applied to the upper end of the floating seal ring to simulate the deformation in Y direction. The assembly simulation retained O-ring freedom until a critical 3 mm gap was achieved between the opposing end faces of the floating ring and seal seat. Subsequently, a uniformly distributed vertical upward pressure load (simulating oil pressure) was applied to the O-ring lower edge. The analysis employed 20 incremental load steps with large deformation enabled.

Floating oil seals are commonly used in rotating equipment such as gearboxes, construction machinery moving parts, etc., and their oil pressure range is first determined by the design pressure of the equipment’s hydraulic or lubrication system. For example, the lubrication system pressure of the walking motor or gearbox of construction machinery is usually in the range of 0.1~0.5 MPa (low-pressure lubrication), while some high-pressure hydraulic systems may reach more than 10 MPa, but floating oil seals are generally used in low-pressure scenarios. To systematically investigate the pressure-dependent behavior of floating oil seals, three representative pressure regimes were established for experimental evaluation, as shown in Table 3: (1) low-pressure conditions (0.5 MPa) representing typical operational scenarios, (2) a medium-pressure range (0.5–3 MPa) for evaluating performance under elevated stress, and (3) a high-pressure environment (5 MPa) to assess the operational limits and failure mechanisms. Comparative performance under these conditions is presented in Table 4.

## 3. Simulation Results Analysis

### 3.1. Contact Analysis of Floating Oil Seals with Assembly Process Effects

**Figure 5:**
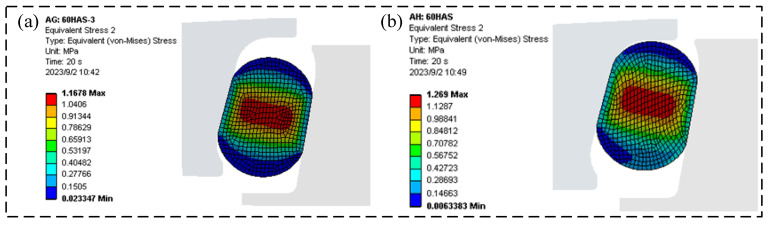
O-ring position considered vs. ignored under no oil pressure and 3 mm clearance: (**a**) without assembly process, (**b**) with assembly process.

**Figure 6:**
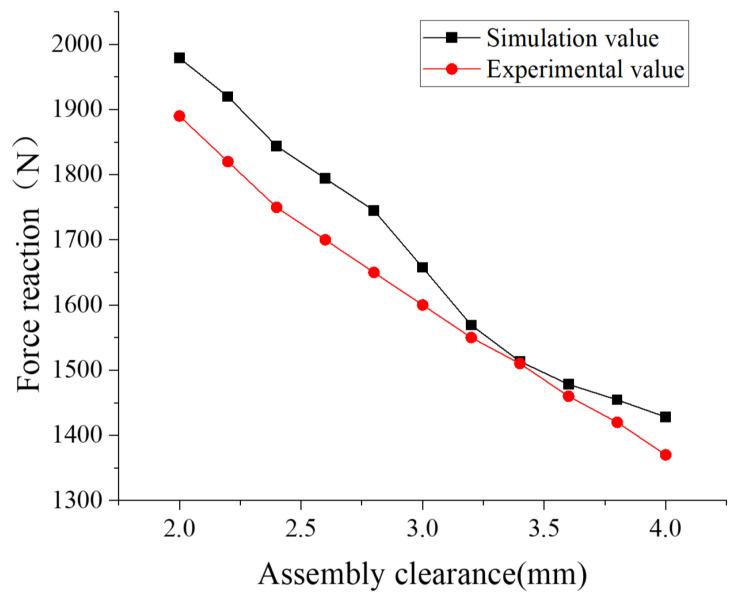
Experimental and simulation comparison of end-face support reaction force under varying assembly clearances.

**Figure 7:**
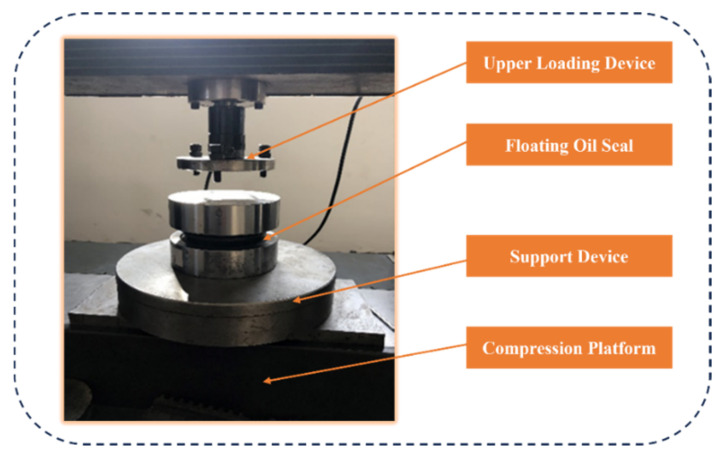
Schematic diagram of the FOS compression test setup.

As shown in Figure 5, accounting for the assembly process revealed that the FOS O-ring failed to reach the relative center position, instead shifting slightly upward. Incorporating the assembly process further increased the maximum von Mises stress (VMS) in the O-ring by 8.7%. Furthermore, the lateral reaction force calculated without considering assembly deviated from the experimental value of 1921 N by approximately 25%, indicating significant error. Consequently, the FOS analysis incorporated the assembly process. Figure 6 presents preliminary measurements of the support reaction force on the floating seal ring end face at various assembly clearances. When the assembly process was accounted for, the simulated end-face support reaction force remained within 5.5% of the experimental value across the clearance range, demonstrating high simulation reliability. Consequently, the floating oil seal contact analysis utilized this assembly-aware methodology. Additionally, Figure 7 schematically illustrates the FOS compression test setup. The diagram details the equipment configuration. During operation, pressure applied via the upper loader induced FOS deformation, supported by the lower fixture, enabling measurement of stress and deformation characteristics.

### 3.2. Effect of Assembly Process on Maximum Von Mises Stress (VMS) in O-Ring

**Figure 8:**
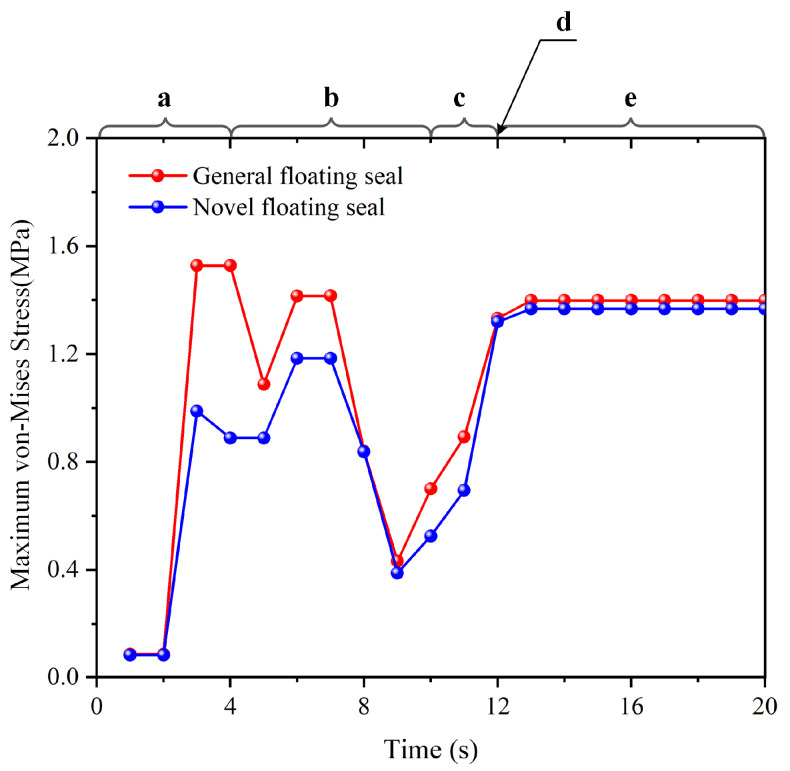
Time-history of O-ring maximum VMS.

**Figure 9:**
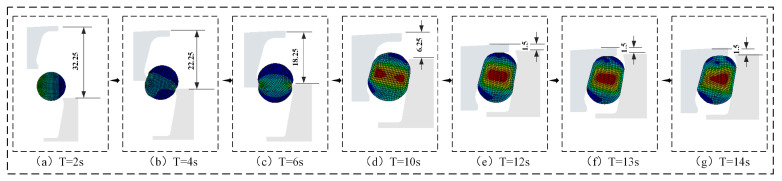
Assembly simulation and VMS evaluation of the FOS O-ring.

**Figure 10:**
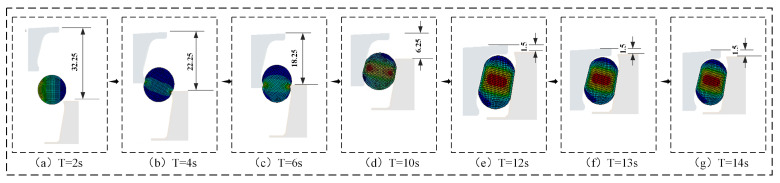
Assembly simulation and VMS evaluation of the NCCFOS O-ring.

The damage criteria for hyperelastic materials were primarily determined by the fourth strength theory in material mechanics [28].

(6) $$\sigma_{s}=\sqrt{\frac{1}{2}[{\left(\sigma_{1}-\sigma_{2}\right)}^{2}+{\left(\sigma_{2}-\sigma_{3}\right)}^{2}+{\left(\sigma_{3}-\sigma_{1}\right)}^{2}]}$$

where $\sigma_{s}$ denotes the VMS. ANSYS implements the fourth strength theory from material mechanics, establishing an allowable stress range of 5.0–20.0 MPa at room temperature. When $\sigma_{s}$ exceeds this range for hyperelastic materials, it accelerates O-ring aging, reduces service life, and compromises seal reliability. Figure 8 shows the temporal evolution of maximum VMS in the O-ring during assembly. Figure 9 and Figure 10 detail the simulated assembly process and resulting VMS distribution under 3 mm assembly clearance and 0.5 MPa oil pressure.

(a) 0 s ≤ T < 4 s: During initial contact between the simulated O-ring and floating seal ring, maximum von Mises stress increased progressively in both conventional and novel floating oil seals due to tightening fit.
(b) 4 s ≤ T < 10 s: As the floating seal ring descended, conventional FOS O-ring stress exhibited a rapid descending–ascending–descending–ascending cyclic trend. Novel NCCFOS O-ring stress showed a stable–rising–stable–falling–rising cycle trend. This stabilized variation reduces wear risk in the novel seal design.
(c) 10 s ≤ T < 12 s: During final positioning between the floating seal ring’s outer cone and seal seat cavity, both seal types showed rapid von Mises stress escalation.
(d) T = 12 s: When the O-ring is precisely located in the inner cavity of the floating seal seat to achieve the state of assembly clearance, the general and novel floating oil seal O-rings will produce radial and axial forces on the floating seal ring, thus achieving an effective floating seal effect.
(e) 12 s < T < 20 s: After the loading of oil pressure, the maximum VMS of the O-ring of the FOS gradually increases and tends to be stable, while that of the NCCFOS O-ring gradually decreases and tends to be stable. This indicates that the novel floating oil seal can distribute stress more effectively under oil pressure loading, thus significantly reducing the maximum VMS of the O-ring.

In summary, simulation results demonstrated that the NCCFOS design achieved both a significantly lower maximum VMS on the O-ring and reduced stress amplitude variation compared to the conventional FOS.

### 3.3. Case 1: Effect of Oil Pressure on the VMS of O-Ring

VMS critically impacts floating oil seals (FOS) through two primary mechanisms: firstly, installation-induced misalignment subjects seals to asymmetric operational loading, accelerating wear and potential failure; secondly, sustained misalignment stress compromises design-condition endurance, significantly reducing service life. Therefore, the long-term performance and longevity of the seal are closely tied to the misalignment stress experienced during installation.

**Figure 11:**
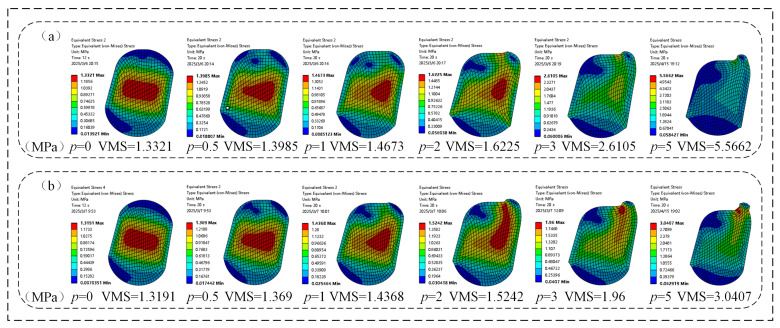
O-ring maximum VMS (**a**) FOS (**b**) NCCFOS.

As shown in Figure 11, both the conventional FOS and NCCFOS exhibited progressively increasing maximum VMS in their O-rings with oil pressure elevation under fixed assembly clearance. At 0 MPa, the maximum VMS of general and novel floating oil seal O-rings was mainly concentrated in the middle of the O-ring, showing a rectangular shape with rounded corners. Compared with a FOS, the maximum VMS of the O-ring in the NCCFOS was reduced by 0.98%. As the oil pressure increased, the maximum VMS of the NCCFOS O-ring was reduced by 2.15%, 2.12%, 6.44%, 33.19%, and 83.06%, respectively. The results show that the increase in oil pressure caused the peak stress area to gradually move closer to the clearance and induce tensile phenomena. Although the NCCFOS did not completely eliminate this issue through optimized design, it slightly reduced the stress, thereby improving its compressive performance.

It can be seen that under the same oil pressure conditions, the NCCFOS better dispersed stress distribution, reduced stress concentration, and improved the durability and reliability of its structure compared to the FOS.

### 3.4. Case 2: Effect of Oil Pressure on Contact Pressure of O-Ring

The contact pressure significantly influences a FOS through two aspects mechanisms. Firstly, under low oil pressure conditions, the floating ring achieves end face contact by relying on the elasticity of the O-ring. Decreasing contact pressure enhances hydrodynamic lubrication conditions by promoting the formation of a more stable oil film, which effectively reduces frictional heating and prevents surface degradation of the sealing interface. Secondly, as the oil pressure increases, the resulting compressive force on the O-ring induces corresponding axial displacement of the floating ring, enhancing the end face contact and establishing a stable oil film. Under medium and high oil pressure conditions, appropriately increasing the contact pressure helps ensure that the sealing ring provides sufficient preload, thereby maintaining a good sealing effect. Therefore, at low oil pressure, the contact pressure should be as low as possible, while at medium to high oil pressure, the contact pressure needs to be moderately increased to ensure the sealing performance of the oil seal and extend its service life.

**Figure 12:**
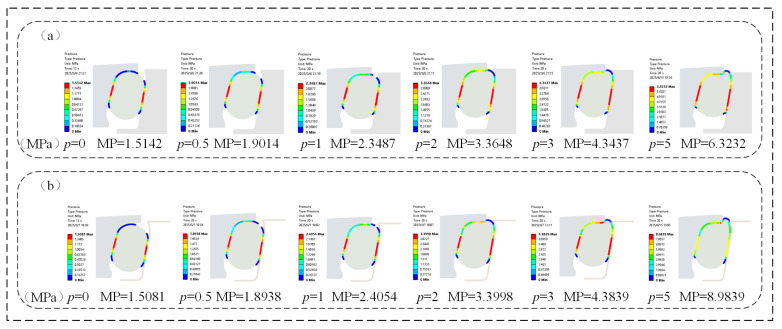
O-ring maximum contact pressure: (**a**) FOS, (**b**) NCCFOS.

As quantitatively demonstrated in Figure 12, under constant assembly clearance conditions, the maximum pressure (MP) of both the FOS and NCCFOS O-rings exhibited positive correlation with increasing oil pressure. The stress distribution profiles reveal a characteristic arcuate pattern, with peak values concentrated in the central region and gradually diminishing toward the periphery. In the absence of oil pressure, compared with the FOS, the maximum contact pressure of the O-ring in the NCCFOS was reduced by 0.40%. With the increase of oil pressure, the maximum contact pressure of the NCCFOS O-ring decreased by 0.39%, −2.41%, −1.04%, −0.90%, and −42.07%, respectively (negative values contact pressure increases). The results show that the NCCFOS better formed an oil film under low oil pressure conditions, avoiding overheating and wear of the sealing surface. Under medium and high oil pressure conditions, an appropriate increase in the contact pressure can effectively improve the sealing performance, prevent leakage, and enhance the sealing effect.

The contact pressure of the NCCFOS was superior to that of the FOS under various oil pressure conditions; especially under high oil pressure, the performance was more significant.

### 3.5. Case 3: Effect of Oil Pressure on O-Ring Contact Friction Stress

The impact of contact friction stress on a FOS is mainly reflected in two aspects. Firstly, under low oil pressure conditions, the oil film has not yet fully formed. The contact area between the floating sealing rings is relatively large, and the contact friction force is relatively high, being dry friction. Excessive friction can cause the sealing surface to overheat and wear, thereby affecting the sealing effect. Secondly, as the oil pressure increases, the lubricating oil gradually forms a stable oil film, the direct contact between the floating sealing rings decreases, and the friction force gradually increases from the oil film lubrication friction. The characteristic of oil film lubrication friction is that the friction force increases, but the oil film effectively isolates the contact surface, reduces direct contact, lessens wear and extends service life. Therefore, at low oil pressure, the contact friction force should be as low as possible to reduce friction and wear. When the oil pressure is high, in order to ensure the sealing performance of the oil seal, the contact friction force should be moderately increased, and its stability and durability can be enhanced through the lubrication friction of the oil film.

**Figure 13:**
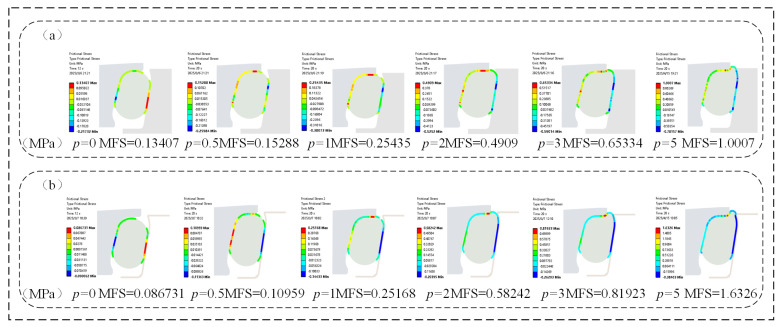
O-ring maximum contact friction stress: (**a**) FOS, (**b**) NCCFOS.

As shown in Figure 13, under a certain assembly clearance, the maximum frictional stress (MFS) of the O-rings in both the FOS and NCCFOS exhibited a positive correlation with increasing oil pressure, accompanied by a proportional expansion of the contact area. In the absence of oil pressure, the NCCFOS design achieved a 54.58% reduction in maximum frictional stress compared to the FOS. Upon pressurization, the NCCFOS O-ring demonstrated a progressive shift in contact pressure behavior, with reductions of 39.50%, 1.06%, −15.71%, −20.24%, and −38.70% at incremental pressure levels (negative values frictional stress increases). These results demonstrate that the NCCFOS maintained low interfacial friction under low-pressure conditions, effectively mitigating wear and thermal overload. At elevated pressures, progressive formation of a stabilized lubricating film transitions friction mechanisms from boundary to hydrodynamic regimes. This lubrication transition enhances sealing integrity while improving operational stability and durability, ultimately extending service life. It can be seen that the new type of NCCFOS can reduce dry friction, and increase oil film lubrication friction, thereby reducing wear and improving sealing performance and service life.

### 3.6. Case 4: Effect of Assembly Clearance on O-Ring Stress and Contact Friction Stress

**Figure 14:**
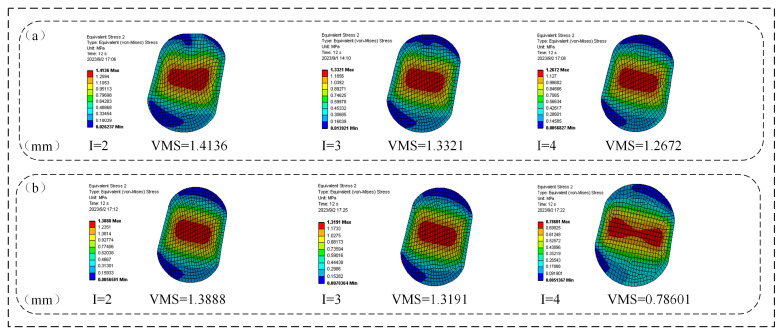
Maximum VMS of O-rings with different assembly clearance: (**a**) FOS, (**b**) NCCFOS.

As illustrated in Figure 14, a larger assembly clearance reduced the maximum VMS on the O-rings of both the FOS and NCCFOS configurations under zero oil pressure. As the assembly clearance decreased, the stress concentration zone on the FOS O-ring progressively shifted towards the interface between the sealing seat and the floating seal. Conversely, the stress concentration area on the NCCFOS O-ring transitioned from a rectangular shape with rounded corners to a dumbbell shape. By optimizing the wear layer, the NCCFOS design achieved reductions in the maximum VMS on its O-ring, thereby enhancing performance and durability. At assembly clearances of 2 mm, 3 mm, and 4 mm, stress reductions of 1.8%, 0.98%, and notably 38% were observed, respectively. These results highlight the NCCFOS’s superior stress dispersion capability.

**Figure 15:**
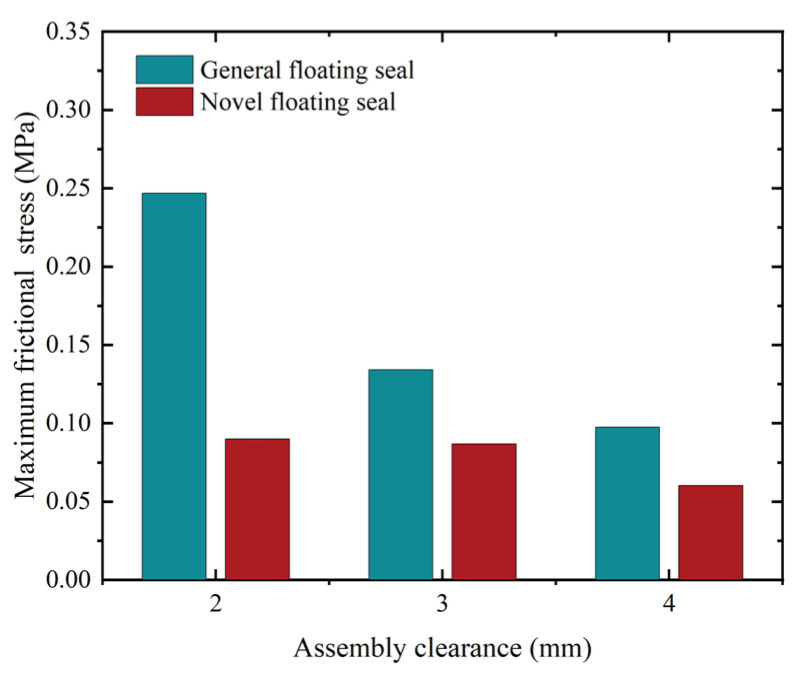
Maximum contact friction stress distribution of O-rings with different clearance.

As shown in Figure 15, under zero oil pressure, the NCCFOS consistently exhibited lower maximum O-ring contact friction stress than the FOS across increasing assembly clearances. This demonstrates the NCCFOS’s effectiveness in reducing O-ring friction under equivalent pressure conditions and minimizing friction and wear across varying clearances. Consequently, the NCCFOS offers improved sealing performance with the potential for extended service life and reduced maintenance costs.

### 3.7. Case 5: Effect of Hardness on VMS and Contact Pressure of O-Rings

**Figure 16:**
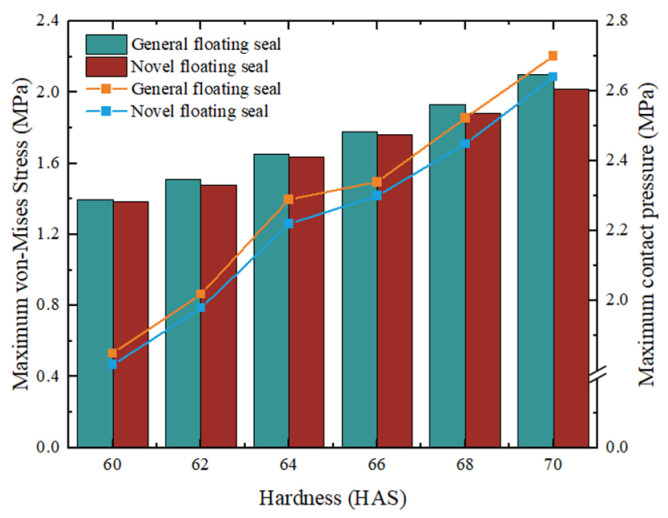
Maximum VMS and contact pressure of O-rings across hardness levels (0.5 MPa oil pressure, 3 mm assembly clearance).

As shown in Figure 16, maximum VMS and maximum contact pressure of O-rings with varying hardness were compared for both FOS and NCCFOS configurations under 0.5 MPa oil pressure and 3 mm assembly clearance. Both stress parameters exhibited a gradual increase with rising O-ring hardness. This indicates that higher hardness corresponds to a more severe stress state in the O-ring, potentially increasing failure risk. Under identical pressure conditions, the NCCFOS O-ring demonstrated lower maximum VMS and contact pressure than its FOS. These results suggest the NCCFOS design enhances O-ring durability and sealing effectiveness under equivalent operational conditions.

## 4. Conclusions

This study introduces an innovative ceramic-coated floating oil seal (NCCFOS) design with superior wear resistance characteristics and evaluates its performance against a conventional FOS through finite element simulation. To evaluate the impact of critical design and operational parameters, parametric analyses were carried out, examining how variations in oil pressure, assembly clearance, and material hardness affected O-ring stress, contact pressure, and frictional stress distribution in the floating seal ring. The findings demonstrate that the NCCFOS design offers substantial improvements in stress distribution, wear resistance, and fatigue performance, providing an effective approach to enhance the operational reliability of coal mining equipment seals. The main conclusions derived from this research are as follows:

1. Incorporating the actual assembly process significantly improved analytical accuracy, particularly in determining the supporting reaction force on the floating seal ring end face. This approach yielded more reliable results compared to methods neglecting assembly considerations.
2. The NCCFOS demonstrated superior performance across various pressure ranges, effectively reducing fatigue accumulation and wear to extend O-ring service life. Particularly under medium-to-high pressure conditions, it enhanced sealing performance while preventing over-compression issues.
3. By optimizing the design, the NCCFOS achieved lower von Mises stress and contact pressure under the influence of oil pressure and O-ring hardness, resulting in improved performance and reliability.
4. Alumina ceramic plates were embedded at the contact between the floating seal ring, the inner wall of the floating seal seat, and the O-ring, and the anti-friction layer design was optimized. Considering the interaction between parameters, this optimized new structure significantly enhanced sealing performance and reduced wear, offering valuable insights for further research.

## Figures and Tables

**Table 1:** Rubber material parameters at varying hardness levels.

Hardness (HSA)	*C*_10_	*C*_01_	*D*_1_	*E* (MPa)
60	0.4825000	0.120624	0.00331600	3.618750
62	0.5229820	0.130745	0.00305800	3.922360
64	0.5679630	0.141991	0.00281700	4.259722
66	0.6182350	0.154559	0.00258800	4.636765
68	0.6747920	0.168698	0.00237100	5.060937
70	0.7388889	0.184721	0.00216542	5.541667

**Table 2:** Parameter setting of FEM for FOS and NCCFOS.

Setting Name	General Model	Novel Model
2D type	Axisymmetric	Axisymmetric
Contact type	Frictional	Frictional and bonded
Friction coefficient	0.2	0.2 and 0.06
Contact algorithm	Augmented Lagrange	Normal Lagrange
Automatic time step	on	on
Large deformation	on	on
Weak spring	off	off

**Table 3:** Typical pressure working range of FOS and NCCFOS.

Pressure Condition	Low Pressure	Medium Pressure	High Pressure
Pressure range (MPa)	0.1~0.5	0.5~3.0	3.0~10

**Table 4:** Simulation research of FOS and NCCFOS under different working conditions.

Working Conditions			Plane183			
Cases	Constant Parameters	Varying Parameters	FOS		NCCFOS	
			Element	Node	Element	Node
Case 1	H = 60 HSA I = 3 mm	*p* = 0, 0.5, 1, 2, 3, 5 MPa	847	2744	893	1041
Case 2						
Case 3						
Case 4	H = 60 HSA *p* = 0 MPa	I = 2, 3, 4 mm				
Case 5	*p* = 0.5 MPa I = 3 mm	H = 60, 62, 64, 66, 68, 70 HSA				

Note: H represents hardness, I represents assembly clearance, and *p* represents oil pressure.

## Data Availability

The original contributions presented in this study are included in the article. Further inquiries can be directed to the corresponding author(s) upon reasonable request.

## Abbreviations

FOS	floating oil seal
NCCFOS	novel ceramic-coated floating oil seal
FEM	finite element model
VMS	von Mises stress
MP	maximum pressure
MFS	maximum frictional stress
